# A randomised controlled study of an audiovisual patient information intervention on informed consent and recruitment to cancer clinical trials

**DOI:** 10.1038/sj.bjc.6603943

**Published:** 2007-09-11

**Authors:** C Hutchison, C Cowan, T McMahon, J Paul

**Affiliations:** 1 Cancer Consultant Nurse, Acute Division, NHS Greater Glasgow and Clyde, Level 3, Office Suite 3B (Room 302), Beatson West of Scotland Cancer Centre, 1053 Great Western Road, Glasgow G12 0YN, UK; 2 Research Practitioner, Beatson West of Scotland Cancer Centre, 1053 Great Western Road, Glasgow G12 0YN, UK; 3 Clinical Trial Coordinator, Cancer Research UK Trials Office, Beatson West of Scotland Cancer Centre, 1053 Great Western Road, Glasgow G12 0YN, UK; 4 Head of Biostatistics, Cancer Research UK Trials Office, Beatson West of Scotland Cancer Centre, 1053 Great Western Road, Glasgow G12 0YN, UK

**Keywords:** cancer clinical trials, patient recruitment, audiovisual patient information, knowledge, understanding

## Abstract

Recruitment to cancer clinical trials needs to be improved, as does patient knowledge and understanding about clinical trials, in order for patients to make an informed choice about whether or not to take part. Audiovisual patient information (AVPI) has been shown to improve knowledge and understanding in various areas of practice, but there is limited information about its effect in the cancer clinical trial setting, particularly in relation to consent rates. In this study, 173 patients were randomised to receive either the AVPI, in addition to the standard trial-specific written information, or the written information alone. There was no difference in clinical trial recruitment rates between the two groups with similar study entry rates: 72.1% in the AVPI group and 75.9% in the standard information group. The estimated odds ratio for refusal (intervention/no intervention) was 1.19 (95% CI 0.55–2.58, *P*=0.661). Knowledge scores increased more in the AVPI group compared to the standard group (*P*=0.0072). The change in anxiety score between the arms was also statistically significant (*P*=0.011) with anxiety improving in the intervention arm more than in the no intervention arm. Audiovisual patient information was shown to be a useful tool in improving patient knowledge and anxiety, but further work is necessary in relation to its effect on clinical trial recruitment rates.

## 

### Clinical trials and recruitment

Clinical trials are the only safe and effective way to improve treatments for cancer. However, less than 5% of patients are recruited to cancer clinical trials every year due to a variety of reasons as discussed in detail by Fayter *et al* (2006). There are factors surrounding the patient, trial and physician with some potentially modifiable, such as issues around the informed consent process and patient refusal. Meeting patients’ information needs and obtaining truly informed consent is challenging in cancer, especially in relation to clinical trials, due to the vulnerability of patients and the complexity of treatments. Misconceptions about clinical trials are common, particularly in relation to randomisation.

Although there are discreet scientific and ethical challenges in each phase of clinical trial, the randomised phase III trial has been shown to be particularly problematic in relation to misinterpretation and poor understanding of patient information, often in relation to the concept of randomisation (Sutherland *et al*, 1990; Lovato *et al*, 1997; Featherstone and Donovan, 2002). Compared with earlier phase studies (Gordon and Daugherty, 2001), relatively high rates of patient refusal have been reported: 28% (Jenkins and Fallowfield, 2000), 40% (Klabunde *et al*, 1999) and 49% (Lara *et al*, 2001). Research undertaken in ‘hypothetical trial’ situations of chemotherapy have shown refusal rates of 40% (Sutherland *et al*, 1990) and 58% (Llewellyn-Thomas *et al*, 1991). Randomised phase III trials comprise the largest component of clinical research in terms of both the number of trials being carried out and also in relation to the number of patients taking part. These trials are therefore of major interest in tackling the worldwide issue of low recruitment to cancer clinical trials.

### Knowledge, understanding and decision making about clinical trials

Knowledge and understanding appear to be related to patient decision making concerning clinical trials, although study findings are conflicting. For example, Simes *et al* (1986) found that patients with a better understanding were less likely to agree to randomisation. However, in another study, Ellis *et al* (2001) found that women who had a better understanding of clinical trial information have more favourable attitudes towards them and are more willing to consider participating.

Fear of randomisation and preference for the doctor to choose the treatment was highlighted as one of the reasons patients’ declined cancer trial participation in the study by Jenkins and Fallowfield (2000). But, as acknowledged by the authors, it is not clear if patients declined because they did not understand the concept of randomisation or because they did, and that evidence from other studies suggest that it is likely to be related to not understanding the concept (Sutherland *et al*, 1990).

### Knowledge and anxiety

Patient anxiety levels are often high due to a number of issues including having a recent cancer diagnosis and cancer treatment (Kelly *et al*, 2002). In most cases, anxiety is part of a normal reaction to cancer. Although appropriate treatment for anxiety is to provide adequate information and support (Kelly *et al*, 2002), the impact on anxiety levels, of increasing knowledge as in the clinical trial situation, is conflicting. Some studies have found that patients with better knowledge and understanding were more anxious (Simes *et al*, 1986), but the majority of studies have found patient anxiety to be unchanged or reduced (Ellis *et al*, 2001).

### Audiovisual patient information

Audiovisual patient information (AVPI) has been shown to improve knowledge and understanding without increasing anxiety (Luck *et al*, 1999; Mason *et al*, 2003), to influence behaviour (Sherafat *et al*, 2003) and to assist patients with decision making (McGregor, 2003). In oncology, video and CD-ROM both have been shown to be acceptable and useful mediums for information transfer (Thomas *et al*, 2000; Agre *et al*, 2002). In the clinical trial setting, video has been shown to improve the consent process (Wirshing *et al*, 2005). However, limited information is available on the effect of AVPI on clinical trial recruitment rates.

Weston *et al* (1997) looked hypothetically at ‘interest in participation’ in a perinatal trial and concluded that a patient information video combined with an information sheet may result in greater participation. However, they acknowledged the hypothetical situation and that it was not possible to know if it translated into improved recruitment for this trial.

Daugherty *et al* (2003) studied the effect of a CD-ROM educational intervention for advanced cancer patients enrolling in phase I and phase II trials and developed an interactive CD-ROM. Patients were randomised to either view the CD-ROM or receive a written information leaflet. Of those who completed the CD-ROM, 71% subsequently enrolled in a trial compared with 58% who received the written information. Although this was a very specific patient population – advanced cancer, and a unique trial setting (phases I and II) – the same issues of vulnerable cancer patients and complex trial information exist for patients considering participation in randomised cancer clinical trials.

Methods such as video, CD-ROM and DVD offer an opportunity to provide generic information about randomised clinical trials to address common misconceptions and misinterpretations, along with cancer site-specific trial information, in an acceptable and effective manner, as a supplement to the written information routinely given to patients considering clinical trials. The limited work that has been carried out in relation to audiovisual aids and clinical trials suggests a positive effect on consent rates, but further work is necessary which led to the impetus for this study.

## PATIENTS AND METHODS

### Study aims

The main aims of the study were

1. to determine the effect of an AVPI intervention on
  - refusal rates to randomised cancer trials
  - knowledge and anxiety and
2. to investigate patients’ perceptions of the AVPI.

Reasons for accepting and declining trial participation were also investigated and will be reported in a future paper.

Ethics approval was obtained from the West Research Ethics Committee.

### Sample

Patients were eligible for the study if they

- had a diagnosis of colorectal, breast or lung cancer and were clinically eligible for entry into a cancer treatment trial randomised against control/standard treatment, or best supportive care,
- had access to a video recorder, CD-ROM or DVD playing facilities and
- could understand English.

Clinical trials for breast, lung and colorectal cancer that involved randomisation against control/standard treatment, or best supportive care, running at the cancer centre, were included in the study.

The primary study end point was the percentage of patients in the study who declined participation in the clinical trial that was presented to them. On the basis of the literature, it was assumed that 40% of patients refuse participation in cancer clinical trials, and in order to have an 80% chance of detecting a 20% difference at the 5% significance level (two-sided), a total of 164 patients were required. This number would detect a reduction in refusal rate from 40 to 20%. This sample size also provides approximately 80% power to detect a moderate standardised difference of 0.45 between continuous variables such as knowledge and anxiety scores.

The study was initially introduced to the patient by their clinician, during explanation of the clinical trial. Following consent, patients were randomised to intervention or no intervention (1 : 1) by contacting the CRUK Clinical Trials Unit (CTU). The study was stratified for individual trial, tumour type, age and sex using the minimisation method, which was implemented in the CTU's Oracle database.

### Data collection and analysis

Data were collected from patient case notes and directly from questionnaires. Patients were seen on two occasions for the purposes of this study. These visits were part of patients’ general medical care, as they were attending the hospital anyway on these days. These were known as ‘visit 1’ (explanation of treatment trial) and ‘visit 2’ (return visit, usually 1 week later, to discuss decision).

#### Measures/instruments

Log sheet: Demographic data on all patients who were approached for the AVPI study were collected and entered into a log sheet. Deprivation status was determined using deprivation categories measured by the Carstairs scores for Scottish postcode sectors (Carstairs and Morris, 1991) using data from the 2001 census (McLoone, 2004).

Clinical Trial Decision Questionnaire: The Clinical Trial Decision Questionnaire is a two-page self-report to assess patients’ reasons for accepting or refusing participation in the trial, perceptions of the consent process and value of the video/CD-ROM/DVD. Patients completed this at visit 2 only. Reasons for accepting/declining the trial were the main focus of this questionnaire. It includes the questionnaire used by Jenkins and Fallowfield (2000) (with kind permission from Dr V Jenkins). Questions in relation to the consent process and usefulness of the video were derived from the literature and consultation with a panel of experts – clinicians and clinical trials nurses – to ensure content validity. The majority of findings from analysis of this questionnaire will be discussed in a future paper focusing on factors affecting decision making in randomised cancer trials.

Knowledge questionnaire: This was a new 12-item questionnaire derived from the literature, patient and professional consultation, for the purpose of this study and has been tested as described in a previous study resulting in slight changes before usage in this study (Hutchison *et al*, 2007). Patients completed this questionnaire at baseline (visit 1) and at visit 2.

Spielberger State and Trait Anxiety Inventory: The Spielberger State and Trait Anxiety Inventory-S assesses anxiety in relation to how one feels at the moment (state). Anxiety was assessed at the same time as knowledge (both visit 1 and visit 2).

#### Intervention

The control arm of the study involved current standard practice in the department for discussing clinical trials with patients. Patients see either a registrar or consultant from the tumour site team, who discusses the trial and administers a trial-specific information sheet and consent form (visit 1). An appointment is then made for them to return to clinic the following week. At this visit (visit 2), they see a clinician from the same team to decide on treatment and whether or not this will be part of a clinical trial.

The intervention consisted of an AVPI tool, which addressed both generic and cancer site-specific clinical trial information, with a particular focus on the concept of randomisation. Areas covered in the AVPI include how drugs/treatments are developed, importance of clinical trials, what randomised trials are and when they are carried out, criteria for taking part, benefits/disadvantages of taking part in a randomised trial, funding issues, voluntariness of decision and freedom to withdraw at any time. ‘Randomisation’ is described by the actress-presenter by using flip charts with pictures of bags of chemotherapy as a visual aid. Within the production, to try to assist in understanding the randomisation concept, several examples of types of randomised trials are given, including a trial where one of the arms is ‘best supportive care’. There are pictures of patients receiving treatment and a voice-over describing the main principles such as that of uncertainty about which treatment is best and also emphasising that the doctor does not decide which treatment the patient will get. At the end of the AVPI, the presenter encourages patients to consider their decision carefully about whether or not to take part, reassuring them that whatever they decide, it will be fully supported by their clinical team. She then refers them to their trial-specific information sheet for further information.

The AVPI was given in addition to standard practice as described in the control arm of the study. The AVPI was developed by clinical and technical staff within the department via a multiprofessional team. Three different versions were made (lung, breast and colorectal cancer) with the same core content, which lasted 10 min in total. Patients were given the AVPI to watch at home: there were three different formats (video, CD-Rom, DVD) to allow for patient preference and availability of equipment at home. The process of developing the AVPI is described in detail elsewhere (Hutchison and McCreaddie, 2007).

#### Analysis

The main comparison of the primary end point between the study arms was performed using logistic regression using all randomised patients. An attempt was made to incorporate minimisation factors used at the time of randomisation into the logistic regression, but the high degree of confounding between gender, tumour type and study meant that of these only gender could be used. The odds ratio was derived from the logistic regression and the *P*-value for the comparison was derived by the likelihood ratio method. The association between baseline patient characteristics and study entry was also assessed in the context of a logistic regression model.

The change in knowledge score from baseline was compared between the two groups via a Mann–Whitney *U*-test (the knowledge score is the number of correctly answered questions expressed as a percentage mark). The statistical significance of within patient changes in knowledge score in each group was assessed using the Wilcoxon signed rank sum test. An assessment of the prognostic value of various baseline characteristics for change in knowledge score was assessed in the context of a linear model incorporating study arm; the dependence of the prognostic value on study arm was assessed by incorporating the appropriate interaction term. Multiple imputation (Rubin, 1987) was applied to assess the robustness of the results of these analyses to missing data. Bootstrap methods were used to estimate the difference in median changes and associated 95% confidence intervals. The association between baseline characteristics and baseline knowledge levels was assessed using the Mann–Whitney *U*-test (2 categories), Kruskal–Wallis test (>2 categories) or, for age, Spearman's rank correlation.

A parallel analysis to that described for the knowledge score was conducted for the anxiety score.

## RESULTS

### Demographic and baseline characteristics

There were 244 patients identified for the AVPI study, of which 13 were not approached as they were considered to be particularly distressed due to being told bad news or experiencing uncontrolled symptoms from their disease. Of the 231 patients who were approached for the study, 173 patients were recruited during the study period of 19 months (January 2005 to August 2006). Two patients were excluded as they did not have access to a video, computer or DVD player. Thus, the refusal rate for this study was 24% (56 out of 229) and included a variety of reasons given by patients, such as they had already made their decision, they felt too upset and they did not want any further information. Of those who refused to take part in this study, only 27% were entered into the clinical trial offered. Patients were entered into a total of 18 different randomised clinical trials during the study period. Although the target number was 164, extra patients were recruited to allow for the proportion of patients for whom the question of trial entry at visit 2 was no longer applicable as they were no longer eligible or, for administrative reasons, the study was no longer available. Baseline characteristics of those recruited to this study were well balanced between the arms, as shown in Table 1.

The majority of patients – 56% (48 out of 86) – chose DVD as their preferred medium for the intervention with 43% (37 out of 86) choosing video and 1% (1 out of 86) CD-ROM.

### Primary end point: clinical trial refusal rate

The primary end point was the proportion of patients refusing clinical trial entry. An intention-to-treat analysis including all patients (and adjusting for baseline minimisation factors age and gender) gives an estimated odds ratio for refusal (intervention/no intervention) of 1.19 (*P*=0.661, 95% CI 0.55–2.58). Although patient refusal was the main reason that patients did not enter clinical trials, 3.5% of patients were then not eligible for the clinical trial and seven patients (4%) did not enter for ‘other’ reasons. These were mainly for reasons of disease progression. Excluding patients who were either not eligible for the trial or could not enter for some other reason gives an odds ratio for refusal of 1.19 (*P*=0.664, 95% CI 0.54–2.60). Table 2 summarises the proportion of patients who subsequently entered into clinical trials.

There were no statistically significant (*P*>0.05) associations between any of the pretreatment patient characteristics as reported in Table 1 and clinical trial entry, nor was there any statistically significant interaction between the study arm and these characteristics. There were no statistically significant differences in the reasons given on the decision questionnaire between the two arms either for those who accepted trial entry or those who refused trial entry.

### Knowledge questionnaire

**Figure 1:**
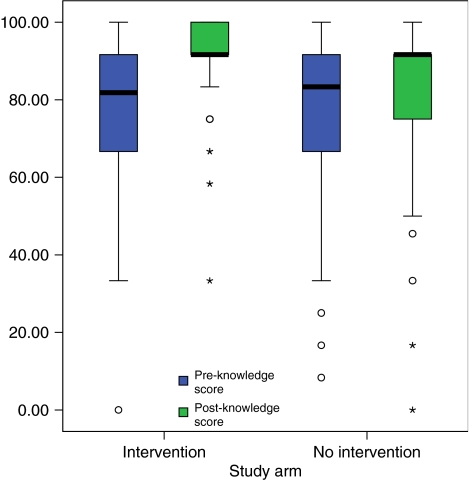
Distribution of percentage knowledge score for patients completing questionnaires at both time points.

In the intervention arm, 81 patients completed the baseline questionnaire and 77 the follow-up questionnaire; the corresponding figures in the no intervention arm are 82 and 77. Seventy-three patients in each arm completed the questionnaire at both time points. The difference in the change in percentage score is statistically significant between the treatment arms (*P*=0.011, *P*=0.0072 (multiple imputation)) with improvements in the knowledge score tending to be higher in the intervention arm. The estimated difference in the median knowledge change score between the groups is 5.0 (95% CI 0.0–16.7). Within both arms, there is a statistically significant improvement in score from pre to post (*P*<0.001 and *P*<0.001 (multiple imputation) in both arms). The distribution of the percentage knowledge score for these patients is shown in Figure 1. Change in knowledge level does not appear to be associated with probability of refusing clinical trial entry.

Age (*P*=0.004), stage (*P*<0.001), friend/family member in research study (*P*=0.015), educational qualifications (*P*<0.001) and tumour type (*P*=0.028) all have statistically significant associations with knowledge at baseline. A multivariate logistic regression (knowledge score dichotomised at the median, variables selected by a forward-stepwise method) was undertaken to examine which pretreatment characteristics were independently associated with baseline knowledge. The outcome of this analysis indicated that education and stage of disease independently were associated with baseline knowledge. Patients who were better educated had higher levels of knowledge (*P*=0.001). Patients who had limited stage of cancer had higher baseline knowledge when compared with patients with advanced cancer (*P*<0.001). Change in knowledge scores did not show any statistically significant association with any of the baseline characteristics in models where the effect of study arm was included, nor was there any indication that the effect of study arm was influenced by any of the baseline characteristics.

### Anxiety questionnaire

In the intervention arm, 77 patients completed the baseline questionnaire and 73 the follow-up questionnaire; the corresponding figures in the no intervention arm are 79 and 69. Sixty-seven patients on the ‘intervention’ arm completed anxiety questionnaires at both time points. On the ‘no intervention’ arm the number was 65. There is a statistically significant difference in anxiety score pretreatment (*P*=0.006), with patients in the ‘intervention’ arm appearing to be more anxious than in the ‘no intervention’ arm.

**Figure 2:**
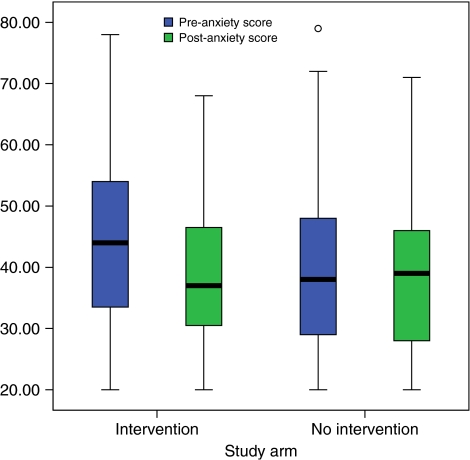
Distribution of percentage anxiety score for patients completing questionnaires at both time points.

The change in anxiety score between the arms is statistically significant (*P*<0.001 and *P*=0.011 (multiple imputation)) with anxiety improving in the ‘intervention’ arm more than in the ‘no intervention’ arm. The estimated difference in the median anxiety change score between the groups is −4.6 (95% CI −7.0 to −2.0). Because of the elevated anxiety in the ‘intervention’ group pretreatment, this means that anxiety levels in the two groups are similar at the ‘post’-assessment. The change from pre to post within the ‘intervention’ group is highly statistically significant (*P*<0.001 and *P*<0.001 (multiple imputation)); there was no statistically significant change in the ‘no intervention’ group (*P*=0.462 and *P*=0.408 (multiple imputation)). The distribution of the anxiety score is shown in Figure 2.

There was no significant association with any of the pretreatment patient characteristics, as reported in Table 1, and anxiety. Change in anxiety scores did not show any statistically significant association with any of the baseline characteristics in models where the effect of study arm was included, nor was there any indication that the effect of study arm was influenced by any of the baseline characteristics.

### Patients’ perceptions of the video/CD-ROM/DVD

Seventy-three patients responded to the questions about their perceptions of the AVPI. Of the patients who received it, 96% (70 out of 73) watched it. Of those who watched it, overall 93% (65 out of 70) found it useful. When asked about the effect the AVPI had on their decision about whether or not to take part in the clinical trial, 42% (25 out of 60) of those who entered the trial said that it had made them want to take part. A large proportion of patients overall stated that the AVPI had no effect on their decision about whether or not to take part in the clinical trial; this was 90% (9 out of 10) of those who refused trial entry and 57% (34 out of 60) of those who entered the trial.

## DISCUSSION

The results showed that although the AVPI had no effect on refusal rates to the randomised cancer trials that the patients were offered, it did have a positive effect on levels of knowledge about clinical trials. It also appeared to reduce anxiety, although there was a statistically significant imbalance in baseline anxiety levels between the two groups, for which we could find no obvious explanation other than the play of chance. In addition, the AVPI was perceived by patients to be a useful adjunct to the informed consent process.

The study was designed with an assumed clinical trial refusal rate of 40% which was based on an average from previous studies (Klabunde *et al*, 1999; Jenkins and Fallowfield, 2000; Lara *et al*, 2001). However, the observed refusal rate for clinical trials in this study of approximately 20% is substantially less than that reported in the literature. Interestingly, the UK study by Jenkins and Fallowfield (2000) reported a refusal rate of 25–28% and, like this study, focused on randomised cancer trials and also had a relatively high proportion of patients with breast cancer.

In our study, the relatively low refusal rate to clinical trials can perhaps be explained within the context of refusal rates for this study where the rate was also low at 24%. Therefore, it could be that the sample was not wholly representative of the population under study. Of those who refused the AVPI study, 73% did not then go into the clinical trial. This is a much higher proportion than the patients who did not enter (for reasons of patient refusal or ineligibility) in both arms of the AVPI study: intervention arm 27.9%; control arm 24.1% (Table 2).

A particular challenge in the informed consent process for clinical trials is to access the patients who have already made their decision about whether or not to take part in the trial, before receiving any information or discussion about it. They are often unwilling to even consider the information, an issue that is difficult in any research study. This would perhaps be easier if AVPI was standard practice in the consent process for clinical trials and was presented to the patient as part of their routine care, although it is acknowledged that there would still be some patients who would choose not to view it.

Garcea *et al* (2005) found differences in attitudes between patients with primary colorectal cancer and patients with secondary colorectal cancer and their willingness to participate in drug trials. They found that more advanced stage patients were more likely to say yes to a clinical trial (56 *vs* 22%). This finding was not substantiated by our study where there were no significant differences in consent rates associated with stage of disease, despite the finding that at baseline, those with limited stage disease were found to be more informed (*P*<0.001). Increase in knowledge did not make people more likely to participate.

A generic approach to information in the AVPI was adopted to focus on the concept of randomization, which was effective in terms of improving patient knowledge and understanding as hoped, but did not improve consent rates. In an attempt to make a generic approach more relevant to the individual patient, the AVPI was customised to tumour type, but it may be that a more specific approach is required. The AVPI could be made specific to an individual trial, although to achieve this would have substantial practical challenges. Wallace *et al* (2006) have shown that in a ‘difficult’ randomised trial with very different treatment options, a multiprofessional education session with patients, which included viewing a customised video, did increase clinical trial consent rates.

An important finding for the cancer trial setting, which is consistent with previous work (predominantly from other specialities), was the effectiveness of AVPI in improving knowledge and understanding without increasing anxiety (Luck *et al*, 1999; Mason *et al*, 2003). It was also encouraging that within both groups, patients were more knowledgeable following the patient information-giving process.

The AVPI was well received, with the majority of patients finding it useful. Only two patients were excluded from the study because they did not have a computer, video or DVD player, which shows that the technology is widely available in patients’ homes. Less than half felt that the AVPI affected their decision about whether or not to take part, which is interesting as other authors have also reported that in decision-making situations, patients relied more on the consultation and the influence/advice of the clinician and less on the supporting tools (Penman *et al*, 1984). However, the literature is conflicting and Garcea *et al* (2005) found, in their study of patients with colorectal cancer, that over 90% claimed to have made their decision after reading the patient information leaflet.

Although the study has provided useful information for practice, its limitations must be acknowledged. It was carried out at a regional cancer centre and findings may not generalise to patients being seen in different settings. The sample consisted of a particularly high number of patients with breast cancer and patients were recruited from a total of 18 different clinical trials. This number of trials could be considered an advantage in terms of generalisability, but could also be seen as a limitation as it was not possible to say with any confidence if there were differences in patients’ decisions between studies, as a result of factors within the trials themselves, for example trials with very different arms where patients may have a preference. Patients’ attitudes were not assessed in this study and it would have been interesting to determine if attitudes changed as a result of the intervention.

In addition, physician interpersonal skills and quality of the interaction have not been addressed in this study. Further work in this area is needed as it has been suggested that these factors may have more of an influence on clinical trial recruitment and that clinical trial acceptance does not appear to be based on a rational model of decision making (Curbow *et al*, 2006). If this is the case, then it is unlikely that consent rates to clinical trials will increase by improving patient knowledge and understanding.

## CONCLUSIONS

Despite the limitations, findings from this study support the use of AVPI as a useful addition to the consent process for randomised cancer trials in terms of improving patient knowledge and understanding before decision making. It appears to reduce anxiety at this time point and has been shown to be an acceptable medium for patients. In this study, AVPI was not shown to have any effect on refusal rates to randomised cancer trials. Further work focusing on AVPI specific to individual trials would be helpful to determine if a more customised approach would be of benefit in relation to clinical trial recruitment.

## Figures and Tables

**Table 1:** Demographic and baseline characteristics

	**Study arm**				**Overall total**	
	**Intervention**		**No intervention**			
	**Col %**	**Count**	**Col %**	**Count**	**Col %**	**Count**
*Gender*						
F	76.7	66	77.0	67	76.9	133
M	23.3	20	23.0	20	23.1	40
Group total	100.0	86	100.0	87	100.0	173

*Tumour type*						
Breast	65.1	56	64.4	56	64.7	112
Colorectal	31.4	27	32.2	28	31.8	55
Lung	3.5	3	3.4	3	3.5	6
Group total	100.0	86	100.0	87	100.0	173

*Age group*						
<50	22.1	19	20.7	18	21.4	37
50–59	23.3	20	24.1	21	23.7	41
60–69	39.5	34	37.9	33	38.7	67
>=70	15.1	13	17.2	15	16.2	28
Group total	100.0	86	100.0	87	100.0	173

*Stage of cancer*						
Limited	68.6	59	66.7	58	67.6	117
Advanced	31.4	27	33.3	29	32.4	56
Group total	100.0	86	100.0	87	100.0	173

*Educational qualifications*						
None	22.2	18	26.3	21	24.2	39
Below degree level	48.1	39	45.0	36	46.6	75
Degree level or higher	29.6	24	28.8	23	29.2	47
Group total	100.0	81	100.0	80	100.0	161

*Previously taken part in research study*						
Yes	8.3	7	15.7	13	12.0	20
No	91.7	77	84.3	70	88.0	147
Group total	100.0	84	100.0	83	100.0	167

*Friend/family member been in research study*						
Yes	12.0	10	12.2	10	12.1	20
No	88.0	73	87.8	72	87.9	145
Group total	100.0	83	100.0	82	100.0	165

*Deprivation status*						
Affluent	27.4	23	27.6	24	27.5	47
Middle	46.4	39	37.9	33	42.1	72
Deprived	26.2	22	34.5	30	30.4	52
Group total	100	84	100	87	100.0	171

**Table 2:** Proportion of patients that subsequently entered into clinical trials

	**Study arm**				**Group total**	
	**Intervention**		**No intervention**			
	**Col %**	**Count**	**Col %**	**Count**	**Col %**	**Count**
*Entered into trial?*						
Yes	72.1	62	75.9	66	74.0	128
No, refused	19.8	17	17.2	15	18.5	32
No, not eligible	2.3	2	4.6	4	3.5	6
No, other	5.8	5	2.3	2	4.0	7
Group total	100.0	86	100.0	87	100.0	173
